# Clinical identification and microbiota analysis of *Chlamydia psittaci-* and *Chlamydia abortus-* pneumonia by metagenomic next-generation sequencing

**DOI:** 10.3389/fcimb.2023.1157540

**Published:** 2023-06-26

**Authors:** Gongxun Xie, Qing Hu, Xuefang Cao, Wenjie Wu, Penghui Dai, Wei Guo, Ouxi Wang, Liang Wei, Ruotong Ren, Yanchun Li

**Affiliations:** ^1^ Department of Pathology, Hunan Provincial People’s Hospital, The First Affiliated Hospital of Hunan Normal University, Changsha, Hunan, China; ^2^ Institute of Innovative Applications, MatriDx Biotechnology Co., Ltd, Hangzhou, Zhejiang, China; ^3^ Foshan Branch, Institute of Biophysics, Chinese Academy of Sciences, Beijing, China

**Keywords:** *Chlamydia psittaci*, *Chlamydia abortus*, Metagenomic next-generation sequencing, Pulmonary microbiome, Chlamydial pneumonia

## Abstract

**Introduction:**

Recently, the incidence of chlamydial pneumonia caused by rare pathogens such as *C. psittaci* or *C. abortus* has shown a significant upward trend. The non-specific clinical manifestations and the limitations of traditional pathogen identification methods determine that chlamydial pneumonia is likely to be poorly diagnosed or even misdiagnosed, and may further result in delayed treatment or unnecessary antibiotic use. mNGS's non-preference and high sensitivity give us the opportunity to obtain more sensitive detection results than traditional methods for rare pathogens such as *C. psittaci* or *C. abortus*.

**Methods:**

In the present study, we investigated both the pathogenic profile characteristics and the lower respiratory tract microbiota of pneumonia patients with different chlamydial infection patterns using mNGS.

**Results:**

More co-infecting pathogens were found to be detectable in clinical samples from patients infected with *C. psittaci* compared to *C. abortus*, suggesting that patients infected with *C. psittaci* may have a higher risk of mixed infection, which in turn leads to more severe clinical symptoms and a longer disease course cycle. Further, we also used mNGS data to analyze for the first time the characteristic differences in the lower respiratory tract microbiota of patients with and without chlamydial pneumonia, the impact of the pattern of *Chlamydia* infection on the lower respiratory tract microbiota, and the clinical relevance of these characteristics. Significantly different profiles of lower respiratory tract microbiota and microecological diversity were found among different clinical subgroups, and in particular, mixed infections with *C. psittaci* and *C. abortus* resulted in lower lung microbiota diversity, suggesting that chlamydial infections shape the unique lung microbiota pathology, while mixed infections with different *Chlamydia* may have important effects on the composition and diversity of the lung microbiota.

**Discussion:**

The present study provides possible evidences supporting the close correlation between chlamydial infection, altered microbial diversity in patients' lungs and clinical parameters associated with infection or inflammation in patients, which also provides a new research direction to better understand the pathogenic mechanisms of pulmonary infections caused by *Chlamydia.*

## Introduction

*Chlamydia* is a family of obligate intracellular Gram-negative bacteria with a unique biphasic life cycle in which a small extracellular infectious basic body (EB) and a metabolically active reticulum (RB) play a special role (Cheong et al., 2019; Marti and Jelocnik, 2022). Taxonomically, *Chlamydia* belongs to the order Chlamydiales and the family Chlamydiaceae and are currently subdivided into more than 10 species (Sachse et al., 2015; Zaręba-Marchewka et al., 2020). They are widely distributed worldwide and infect a variety of hosts, including amoebae, insects, aquatic animals, reptiles, birds and mammals. *C. trachomatis* and *C. pneumoniae* have been described as causative agents of female genital tract infections or trachoma and respiratory tract infections, respectively. In addition, other species such as *C. abortus, C. psittaci, C. pecorum and C. suis* are also thought to play a major role in animal infections (Cheong et al., 2019).

*C. psittaci* and *C. abortus* are known for their zoonotic potential and are often thought to be responsible for nautilus disease and human abortion, respectively (Pospischil et al., 2002; Wu et al., 2021). Human infection with *C. psittaci* is mainly through inhalation of dust containing respiratory secretions or dried faeces, or through direct contact with fresh faeces from infected birds or poultry (Harkinezhad et al., 2007; Shaw et al., 2019; Qin et al., 2022). There have also been some reports of possible human-to-human transmission of *C. psittaci* (Wallensten et al., 2014; Zhang et al., 2022c). Notably, as a newly discovered chlamydial species, *C. abortus* is closely related to the well-known *C. psittaci* in terms of genetics, host relatedness and associated disease pathological features (Joseph et al., 2015). However, to date, only sporadic cases of pneumonia caused by *C. abortus* have been reported worldwide (Ortega et al., 2015; Imkamp et al., 2022; Zhu et al., 2022a). *C. abortus* can be transmitted to humans *via* urine, faeces, milk, amniotic fluid, placenta, aborted foetuses and other excretion routes in sick animals (Zhu et al., 2022a).

Patients with chlamydial pneumonia usually present with non-specific clinical signs such as fever and weakness, with occasional loss of appetite, cough, myalgia and headache (Tang et al., 2022). Currently, the taxonomic identification of *Chlamydia* is based on isolation and culture of the pathogen, serological testing and further molecular typing (e.g. polymerase chain reaction and 16S rRNA gene sequencing) (Corsaro and Greub, 2006). *Chlamydia* culture usually requires the rigour of an experienced professional in a Class C3 laboratory. There are commercially available antigen or antibody test kits for *Chlamydia*, but the common cross-contamination of serological tests can also lead to false positive results (Corsaro and Venditti, 2004). Relative to culture and serological assays, PCR or PCR-based molecular typing techniques have better efficacy for *Chlamydia* detection or species identification, but require pre-assumptions and primer design specific to the chlamydial genome (Barati et al., 2017). In summary, the non-specific clinical presentation and the many limitations of pathogen identification methods dictate that chlamydial pneumonia due to *C. psittaci* and *C. abortus* is likely to be poorly diagnosed or even misdiagnosed, and may further result in delayed treatment or unnecessary antibiotic use.

Recently, metagenomic next-generation sequencing (mNGS) has emerged as a novel and promising method for the detection of infectious agents. Based on non-preference and high sensitivity, mNGS provides more sensitive pathogen detection results than conventional culture, especially for rare pathogen detection involved in clinically challenging cases (Miao et al., 2018; Li et al., 2021a; Wang et al., 2021; Yin et al., 2022). However, existing studies are mainly focus on the applicability of mNGS for the detection of *Chlamydia* infection in case reports or diagnostic performance assessments (Chen et al., 2020b; Duan et al., 2022; Liu et al., 2022b; Tang et al., 2022; Yang et al., 2022; Yin et al., 2022; Zhang et al., 2022c; Zhu et al., 2022b), but not on the microbiome characteristics of the lower respiratory tract in patients with chlamydial pneumonia. In the present study, we first investigated the pathogenic profile of pneumonia patients with different chlamydial infection patterns, and subsequently compared the microbiota differences in the lower respiratory of patients with and without chlamydial pneumonia, and also analyzed the impact of chlamydial infection patterns on the microbiota dynamics of lower respiratory tract and finally we explored the correlation between the characteristics and the clinical parameters of patient care. Our results may answer to some extent the relevance of the lower respiratory microbiota to the signs and course of chlamydial pneumonia and also provide new insights into the pathogenesis of infectious pneumonia due to *Chlamydia*.

## Materials and methods

### Patient enrollment

The present study retrospectively enrolled patients with suspected pulmonary infection from January to December 2021 at Hunan Provincial People’s Hospital, The First Affiliated Hospital of Hunan Normal University. Demographic information, clinical symptoms, laboratory test results, imaging examination results, diagnosis and treatment history, clinical course of the disease and outcomes were collected from electronic medical records.

This study was approved by the Ethics Committee of the Hunan Provincial People’s Hospital and conducted in accordance with Declaration of Helsinki principles and relevant ethical and legal requirements.

### Clinical sample collection and DNA extraction

Bronchoalveolar lavage fluid (BALF), sputum or peripheral blood samples were collected from each patient with the consent of themselves or their surrogates. The BALF samples were collected by experienced bronchoscopists after anesthesia with midazolam. The peripheral blood was centrifuged at 1600 *×g* for 10 min and the collected supernatant was further centrifuged at 16000 *×g* for 10 min to separate plasma. DNA extraction and library preparation from clinical samples were performed by using an NGS Automatic Library Preparation System (MatriDx Biotech Corp. Hangzhou). The quality of DNAs was assessed using a BioAnalyzer 2100 (Agilent Technologies; Santa Clara, CA, United States) combined with quantitative PCR to measure the adapters before sequencing.

### Metagenomic next-generation sequencing

Qualified DNA libraries were pooled together and subsequently sequenced on Illumina NextSeq500 system (50 bp single-end; San Diego, CA, United States). To control the quality of each sequencing run, a negative control and a positive control were conducted in parallel. A total of 10 - 20 million reads were generated for each sample. The raw sequenced reads were first processed with quality control to remove short (length < 35 bp), low quality and low complexity reads, as well as those corresponding to adapters. Host sequences were filtered out based on the alignment to the human-secific database in NCBI using Bowtie2 (version 2.3.5.1). The clean reads were thus aligned to a manual-curated microbial database using Kraken2 (version 2.1.2; confidence = 0.5) for quick taxonomic classification. The classified reads of interested microorganisms were further validated through a second alignment to the microbial database using Bowtie2. The classification of candidate reads might also be conducted by BLAST (version 2.9.0) whenever the results of Kraken2 and Bowtie2 were inconsistent.

Before data analysis, microbes detected in clinical samples were first compared with those detected in the parallel NTC (no template control). Microorganisms with a reads per million (RPM) above 10 or if the organism was not detected in the parallel NTC were maintained and defined as microbiota for further analysis. Substantially, all species of microbiota were looked up in PubMed to determine whether the organisms cause pneumonia and the positive pathogenic microorganisms were defined as pathogens.

### Statistical analysis

Categorical variables, shown as frequencies and percentages, were compared using Fisher’s exact test. Continuous measurement data following normal distribution were shown as mean ± standard deviation (x ± s), and non-normal distribution was shown by median (range). Differences and significance were calculated using the Wilcoxon test or Kruskal-Wallis test (for non-normal distribution data). SPSS 26.0 (IBM Corporation) was used for statistical analysis. Data visualization was performed in R (Version 4.2.1). Specifically, bivariate or multivariate difference analysis was performed using unsupervised clustering methods with reference to the core steps of limma, voom, fit, eBays, etc. The final similarities (or differences) between groups were demonstrated using plotMDS in the limma package and the results were output using the topTable method sorted by *P*-value. RPKM values of microbes were log2 transformed before their relative abundance was analyzed. Variations in composition and abundance of microbes between groups were analyzed using the limma package. In this study, two-sided *P* values < 0.05 were considered statistically significant. Particularly, for multiple comparisons, FDR method was used to correct the primary *P*-value.

## Results

### Demographic and clinical characteristics of the cohort

**Figure 1:**
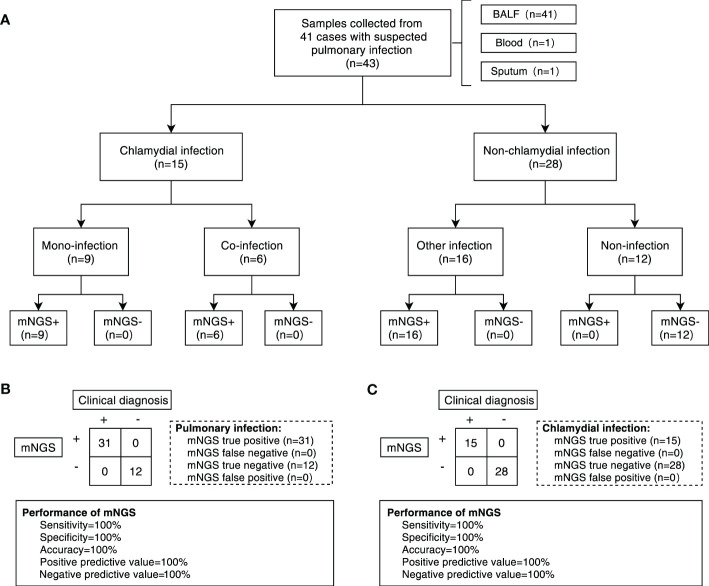
Overall design of the study and diagnostic performance of mNGS test. **(A)** Flowchart of patients and samples classification. A total of 43 samples were obtained from 41 patients for our study. Samples were divided into chlamydial infection and non-chlamydial infection groups. Samples in chlamydial infection were further divided into chlamydial mono-infection (CP group and CA group) and co-infection (PA group) subgroups, and samples in non-chlamydial infection group were further divided into other infection (O group) and non-infection (N group) subgroups. **(B)** The performance of mNGS in diagnosing pulmonary infection based on clinical diagnosis. **(C)** The performance of mNGS in diagnosing chlamydial infection based on clinical diagnosis. BALF, bronchoalveolar lavage fluid. mNGS, metagenomic next-generation sequencing.

A total of 43 samples from 41 patients, i.e. 23 (53.5%) females and 20 (46.5%) males, were included according to the sample entry exclusion criteria for this study, with a median age of 55 years (range 30 - 74 years) and a median length of hospital stay of 12 days (range 4 - 47 days) (**Table 1**). Among these enrolled samples, a total of 25 (58.2%) had underlying diseases (**Table 1**). These clinical samples were first divided into the chlamydial pneumonia group (n=15) and the non-chlamydial pneumonia group (n=28) based on whether the pathogenic microorganism identified by mNGS contained *Chlamydia*. The two groups mentioned above were further divided into 5 subgroups according to clinical diagnosis. In details, the chlamydial pneumonia group was subdivided into the *C. psittaci* mono-infection subgroup (CP, n=7), the *C. abortus* mono-infection subgroup (CA, n=2) and the *C. psittaci*-*C. abortus* co-infection subgroup (PA, n=6). The non-chlamydial pneumonia group was subdivided into the infectious subgroup of other pathogens (O, n=16) and the non-infectious subgroup (N, n=12) (**Figure 1A**).

**Table 1:** Demographic and clinical characteristics of patients and samples.

Characteristics	Overall (n=43)	CP (n=7)	CA (n=2)	PA (n=6)	N (n=12)	O (n=16)	*P*-value
Demographics							
Age, median (range, years)	55 (30-74)	53 (30-68)	59.5 (55-64)	57.5 (36-74)	52.5 (32-73)	59 (30-71)	0.655
Gender, (n, %) **							0.004
Female	23 (53.5)	7 (100.0)	0	5 (83.3)	6 (50)	5 (31.3)	
Male	20 (46.5)	0	2 (100.0)	1 (16.7)	6 (50)	11 (68.7)	
Temperature, median (range,°C) **	37.5 (36.0-41.0)	39.5 (39.0-40.0)	39.5 (39.0-40.0)	39.8 (38.5-41.0)	36.6 (36.2-39.0)	36.5 (36.0-39.0)	<0.001
Contact history (n, %) *	4 (9.3)	2 (28.6)	1 (50.0)	1 (16.7)	0	0	0.016
Underlying disease (n, %)	25 (58.2)	5 (71.4)	2 (100.0)	4 (66.7)	4 (33.3)	10 (62.5)	0.304
Clinical manifestations (n, %)							
Fever **	28 (65.1)	7 (100.0)	2 (100.0)	6 (100.0)	6 (50.0)	7 (43.8)	0.009
Cough	30 (69.8)	7 (100.0)	2 (100.0)	5 (83.3)	8 (66.7)	8 (50)	0.123
Expectoration	23 (53.5)	5 (71.4)	2 (100.0)	3 (50.0)	6 (50.0)	7 (43.8)	0.613
Chills **	9 (20.9)	3 (42.8)	2 (100.0)	2 (33.3)	1 (8.3)	1 (6.3)	0.009
Weakness **	15 (34.9)	7 (100.0)	1 (50.0)	5 (83.3)	0	2 (12.5)	<0.001
Myalgia *	3 (7.0)	2 (28.6)	0	1 (16.7)	0	0	0.039
Diarrhea	2 (4.7)	0	0	1 (16.7)	0	1 (6.3)	0.53
Urinary and fecal incontinence *	3 (7.0)	2 (28.6)	0	1 (16.7)	0	0	0.039
Poor mental state *	19 (44.2)	5 (71.4)	2 (100.0)	5 (83.3)	3 (25.0)	4 (25.0)	0.011
Poor appetite **	19 (44.2)	6 (85.7)	2 (100.0)	5 (83.3)	3 (25.0)	3 (18.8)	0.001
Poor sleep	10 (23.3)	2 (28.6)	0	4 (66.7)	2 (16.7)	2 (12.5)	0.106
Anhelation **	7 (16.3)	4 (57.1)	1 (50.0)	0	0	2 (12.5)	0.007
Headache	4 (9.3)	1 (14.3)	0	2 (33.3)	0	1 (6.3)	0.199
Dizzy *	4 (9.3)	2 (28.6)	0	2 (33.3)	0	0	0.019
Nausea and vomiting	3 (7.0)	2 (28.6)	0	0	0	1 (6.3)	0.228
Painful throat	2 (4.7)	1 (14.3)	0	1 (16.7)	0	0	0.178
Conscious disorders *	6 (14.0)	3 (42.8)	0	2 (33.3)	0	1 (6.3)	0.043
Chest tightness	2 (4.7)	1 (14.3)	0	1 (16.7)	0	0	0.178
Palpitations	2 (4.7)	1 (14.3)	0	1 (16.7)	0	0	0.178
**Complications (n, %)** **	7 (16.3)	0	1 (50.0)	4 (66.7)	0	2 (12.5)	0.002
Laboratory examination, mean (range)							
WBC (3.5–9.5 × 10^9/L) *	9.5 (2.7-21.0)	5.2 (4.2-6.5)	11.8 (9.5-14.2)	9.2 (6.4-12.4)	10.5 (4.2-21.0)	11.3 (2.7-19.0)	0.044
NE% (45–75%)	82.0 (0.8-96.1)	87.3 (0.8-88.4)	83.1 (82.4-83.7)	91.5 (0.9-96.1)	76.6 (40.1-94.7)	87.7 (1.7-93.7)	0.247
CRP (<10mg/L) **	139.3 (4.0-249.0)	139.3 (67.1-164.0)	159.2 (93.3-225.0)	203.3 (190.9-249.0)	41.6 (12.5-169.8)	109.1 (4.0-236.9)	0.006
PCT (0.02–0.05ng/mL)	0.32 (0.02-44.0)	0.54 (0.14-13.00)	0.39 (0.12-0.66)	0.44 (0.20-2.23)	0.36 (0.03-9.37)	0.24 (0.02-44.00)	0.845
IL-6 (0-7pg/mL)	65.6 (8.0-518.1)	92.0 (8.6-108.1)	N.A.	75.2 (8.0-518.1)	53.7 (11.7-68.4)	178.1 (21.4-373.8)	0.67
ESR (0-20mm/h)	60.0 (6.0-119.0)	62.0 (49.0-98.0)	65.5 (60.0-71.0)	38.0 (25.1-92.0)	30.0 (23.0-99.0)	64.0 (6.0-119.0)	0.532
D-D (0-0.55mg/L) *	2.8 (0.3-325.0)	4.4 (2.8-7.5)	1.9 (1.7-2.1)	2.4 (1.0-6.7)	1.5 (0.7-3.2)	5.2 (0.3-325.0)	0.025
BNP (0-125pg/mL)	508.6 (28.9-6430.0)	804.3 (273.2-6430.0)	498.6 (498.6-498.6)	590.8 (141.9-1028.1)	431.1 (28.9-785.0)	440.4 (77.0-2295.9)	0.72
K (3.5-5.3mmol/L) *	3.3 (2.9-4.4)	3.1 (3.0-3.2)	3.9 (3.3-4.4)	3.4 (3.1-4.3)	3.3 (3.2-4.1)	3.6 (2.9-4.4)	0.016
Na (137-147mmol/L)	137.5 (116.0-159.0)	138.0 (136.0-141.0)	124.0 (116.0-132.0)	130.0 (124.6-138.0)	138.0 (129.0-145.0)	141.0 (129.7-159.0)	0.058
Cl (99-110mmol/L)	101.0 (78.0-122.0)	106 (96.0-109.0)	84.5 (78.0-91.0)	97.2 (85.9-106.0)	101.0 (95.0-102.0)	105.0 (93.0-122.0)	0.059
Ca(2.10-2.90mmol/L)	1.99 (1.04-2.47)	1.99 (1.75-2.47)	2.18 (2.08-2.27)	1.88 (1.70-2.07)	1.99 (1.95-2.02)	1.57 (1.04-2.08)	0.228
LDH (100-240U/L)	317.4 (121.9-561.5)	336.0 (296.6-561.5)	357.0 (232.9-481.0)	384.0 (261.9-461.0)	287.4 (121.9-339.5)	306.8 (256.0-392.3)	0.427
CK (10-175U/L)	159.0 (11.0-2472.8)	327.9 (56.3-2472.8)	151.6 (111.5-191.6)	159.0 (47.2-1218.9)	303.4 (32.0-1464.3)	88.0 (11.0-1254.0)	0.871
MYO (0-85ng/mL)	88.1 (24.4-1395.7)	60.4 (24.4-523.3)	130.7 (82.1-179.2)	99.0 (61.0-111.2)	347.2 (24.6-1079.5)	88.1 (47.2-1395.7)	0.71
CTnl (0-0.034ng/mL)	0.025 (0.003-0.097)	0.025 (0.003-0.039)	0.031 (0.024-0.037)	0.009 (0.005-0.019)	0.095 (0.092-0.097)	0.025 (0.005-0.071)	0.134
ALT (9-50U/L)	56.9 (10.4-619.9)	64.3 (21.8-97.3)	77.5 (34.7-120.3)	94.0 (27.3-177.0)	27.6 (10.4-619.9)	34.1 (17.3-272.0)	0.728
AST (15-40U/L)	68.6 (12.0-857.3)	55.4 (22.2-206.3)	123.3 (29.2-217.4)	91.7 (33.6-217.0)	16.3 (12.9-857.3)	14.1 (12.0-171.8)	0.262
BUN (1.7-8.3mmol/L)	6.4 (1.7-19.4)	3.2 (2.2-6.4)	4.87 (3.1-6.6)	4.4 (1.7-7.6)	6.4 (4.6-8.4)	10.7 (2.8-19.4)	0.122
CREA (40-100μmol/L)	66.5 (15.0-143.0)	66.5 (52.0-69.4)	73.0 (57.0-89.0)	60.1 (35.2-76.1)	88.0 (15.0-132.4)	64.0 (25.8-143.0)	0.882
**Hospitalized days, median (range)**	12 (4-47)	11 (6-29)	12.5 (12-13)	9.5 (8-13)	12 (4-29)	14.5 (7-47)	0.811
**Outcome (n, %)**							0.636
Improved	35 (81.4)	5 (71.4)	2 (100.0)	4 (66.7)	11 (91.7)	13 (81.3)	
Not improved	8 (18.6)	2 (28.6)	0	2 (33.3)	1 (8.3)	3 (18.8)	

P.S., Significant differences among groups are indicated by asterisks, with * represents p<0.05 and ** represents p<0.01. N.A., Not applicable.

The majority of clinical symptoms in the enrolled patients were fever (65.1%), cough (69.8%), sputum (53.5%) and other non-specific symptoms (**Table 1**). The incidence of various clinical symptoms was significantly different across subgroups and patients with chlamydial infection displayed a relatively higher percentage, including fever (*P* = 0.009), chills (*P* = 0.009), weakness (*P* < 0.001), muscle aches (*P* = 0.039), urinary incontinence (*P* = 0.039), poor mental status (*P* = 0. 011), loss of appetite (*P* = 0.001), anhelation (*P* = 0.007), dizziness (*P* = 0.019) and impaired consciousness (*P* = 0.043). Clinical test data were also significantly different among subgroups, such as WBC (*P* = 0.044), CRP (*P* = 0.006) and K^+^ (*P* = 0.016) as listed in **Table 1**. After clinical treatment, most cases (81.4%) were found to be improved. However, complications occurred in seven patients during hospitalization and four of them were from the PA subgroup. Notably, based on previous clinical and public health management experience, the primary risk factor for chlamydial pneumonia is exposure to chickens or domestic animals, but in this study, only four patients with chlamydial pneumonia had a clear history of poultry or domestic animal contact, indicating that there might have other source of infection which is responsible for the occurrence of chlamydial pneumonia in this region. As shown in **Table 1**, these four patients were assigned to the CP group (2 patients), CA group (1 patient) and PA group (1 patient), while the risk factor exposure was not included as a grouping parameter in the subsequent microbiome difference analysis due to the small subset of these patients.

### mNGS can effectively assist in the clinical diagnosis of chlamydial pneumonia

The methodological performance of mNGS in the diagnosis of chlamydial pneumonia was assessed by the receiver operating characteristic (ROC) curve and the final clinical diagnosis was included as the assessment criterion. In this study, the overall performance of mNGS in the detection of pathogens in the total enrolled samples obtained a sensitivity of 100%, an accuracy of 100% and a positive predictive value of 100% (**Figure 1B**). Meanwhile, the sensitivity, specificity, accuracy, positive predictive value and negative predictive value of mNGS for detecting *Chlamydia* infection were all 100% (**Figure 1C**). For both *C. abortus* and *C. psittaci*, the area under the ROC curve (AUC) for mNGS was 1. The cut-off values calculated from the Youden index for *C. abortus* and *C. psittaci* were 0.115 and 0.080, respectively (**Supplementary Figure 1**).

### Spectrum analysis of responsible pathogens in different subgroups of *Chlamydia* infection

**Figure 2:**
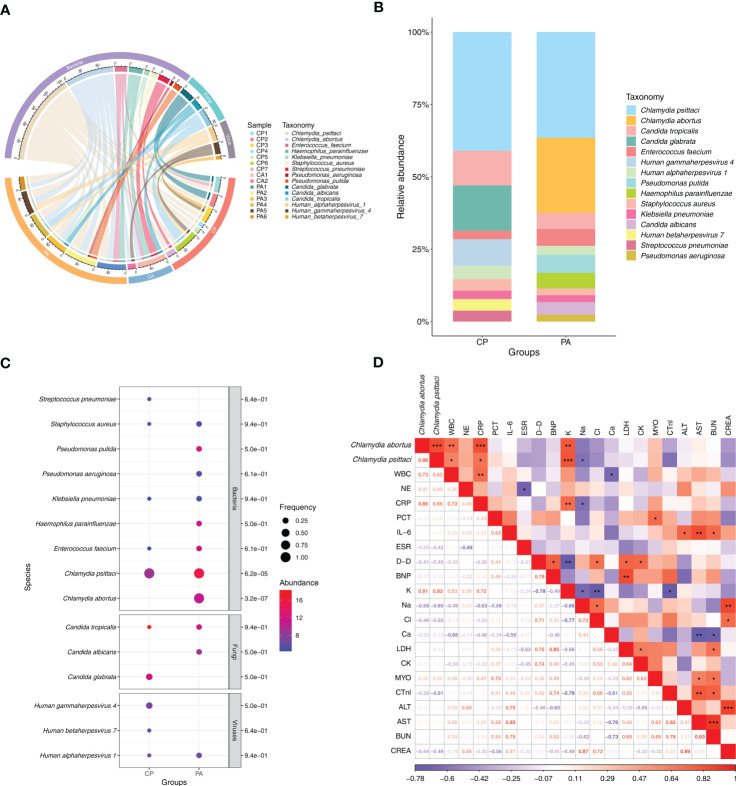
Distribution of responsible pathogens in patients with chlamydial pneumonia and analysis of the association between responsible pathogens and clinical indicators. **(A)** Composition of responsible pathogens in the different samples. **(B)** Relative abundance of responsible pathogens between different chlamydial pneumonia subgroups. **(C)** Differences in relative abundance and detection frequency of responsible pathogens between chlamydial pneumonia subgroups. *P* value were listed on the right of the bubble plot. Red and blue indicate high and low abundance respectively, and the bubble size reflects the frequency of microbes detected. **(D)** Spearman correlation analysis between responsible pathogens with clinical data. Red values indicate species were positively correlated with clinical data, while blue ones indicate the species were negatively correlated with clinical data. Significant associations (*P*<0.05) are indicated by asterisks, with * represents *P*<0.05, ** represents *P*<0.01 and *** represents *P*<0.001. WBC, white blood cell; NE, neutrophil; CRP, C-reactive protein; PCT, procalcitonin; IL-6, interleukin-6; ESR, erythrocyte sedimentation rate; D-D, D-Dimer; BNP, brain natriuretic peptide; K, potassium ions; Na, sodium ion; Cl, chloride ion; Ca, calcium ion; LDH, lactic dehydrogenase; CK, creatine kinase; MYO, Myoglobin; CTnl, cardiac troponin I; ALT, alanine transaminase; AST, aspartate transferase; BUN, blood urea nitrogen; CREA, creatinine.

In this study, clinical samples identified as chlamydial infection using mNGS included 13 cases of BALF, 1 case of sputum, and 1 case of peripheral blood. A total of 15 responsible pathogens were detected and more than one pathogen was detected in each clinical sample (**Figure 2A**; **Supplementary Table 1**). We found that different subgroups of *Chlamydia* infection had different responsible pathogen profiles, with 11, 6, and 11 responsible pathogens detected in the CP, CA, and PA groups, respectively (**Figure 2A**; **Supplementary Table 1**). The relative abundance of the responsible pathogens also differed significantly among different chlamydial pneumonia subgroups. As shown in **Figure 2B**, the CP and PA groups have different spectrum of responsible pathogens. Combined with Bubble matrix analysis (**Figure 2C**), we can find that the main responsible pathogens identified in the CP group were *C. psittaci* (Frequency: 100% (7/7), Relative abundance: 41.0%) and *Candida glabrata* (28.6% (2/7), 15.7%). As expected, *C. psittaci* (100% (5/5), 36.5%) and *C. abortus* (100% (5/5), 25.9%) were the most enriched species in the PA group. In addition, the relative abundance of the following two responsible pathogens was significantly different (*P* < 0.05) among the two subgroups: *C. abortus* (0.0%, 25.9%) and *C. psittaci* (41.0%, 36.5%) (**Supplementary Table 2**).

### Correlation analysis between responsible pathogens and clinical indices of *Chlamydia*-infection patients

To explore the correlation between clinical characteristics and responsible pathogens in chlamydial infection, spearman test was performed on responsible pathogens mentioned above (**Figure 2D**). The results showed that the abundance of *C. abortus* was significantly and positively correlated with the dynamics of clinical indicators such as white blood cell count (R = 0.73, *P* = 0.007), C-reactive protein (CRP) (R = 0.85, *P* = 0.001) and potassium ion content (K^+^) (R = 0.81, *P* = 0.001), but negatively correlated with sodium ion content (Na^+^) (R = -0.58, *P* = 0.064) (**Figure 2D**; **Supplementary Figures 2E–H**). *C. psittaci* was significantly and positively correlated with white blood cell count (R = 0.62, *P* = 0.032), CRP (R = 0.69, *P* = 0.013) and K^+^ (R = 0.83, *P* = 0.001) and significantly negatively correlated with sodium ion content (Na^+^) (R = -0.6, P = 0.05) (**Figure 2D**; **Supplementary Figures 2A–D**).

### Characterization of pulmonary microbial communities in different subgroups of *Chlamydia* infection

**Figure 3:**
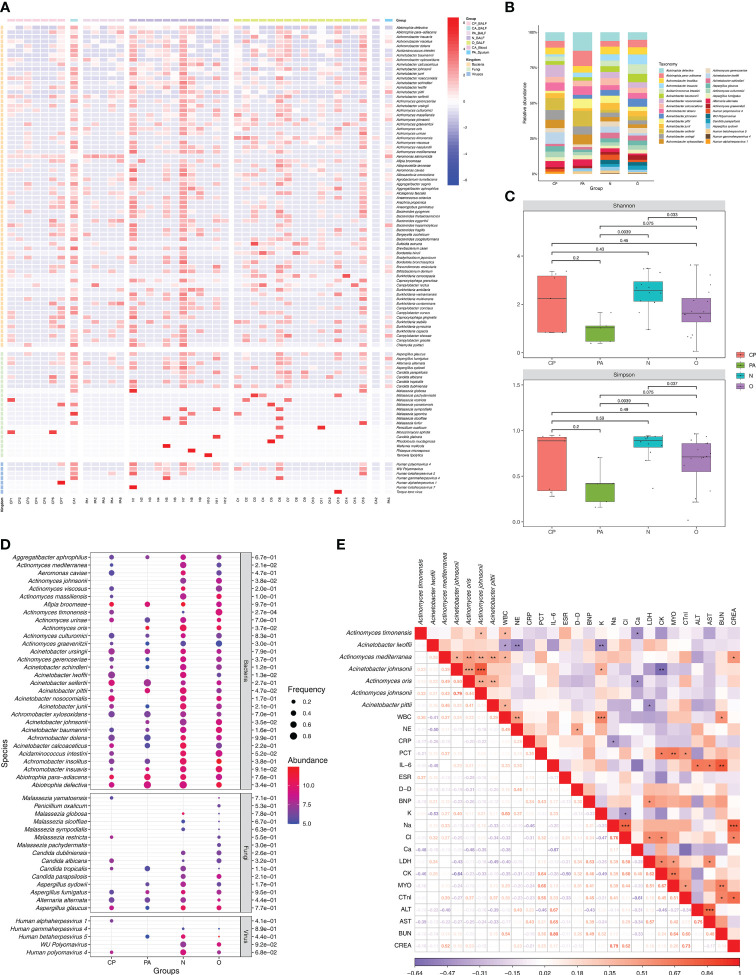
Microbiota communities at species level between different groups and analysis of the association between lung microbiota species and clinical data. **(A)** Composition of microbiome in the different samples. The heatmap exhibited the top 100 frequent species of the top 70 bacteria and all fungi and viruses. Group names and microbial kingdom are indicated by color bars on the right of map. **(B)** Relative abundance of frequency ranking top 30 microbes (consisted of the top 20 bacteria, the top 5 fungi and the top 5 viruses) in 40 BALF from different groups. **(C)** Alpha diversity differences of lung microbiota communities at species level between groups. Each dot represents one sample from each group. **(D)** Differences in relative abundance and detection frequency of frequency ranking top 50 microbes (consisted of the top 30 bacteria, the top 15 fungi and the top 5 viruses) in 40 BALF from different groups. *P* value were listed on the right of the bubble plot. Red and blue indicate high and low abundance respectively, and the bubble size reflects the frequency of microbes detected. **(E)** Spearman correlation analysis between microbial species with clinical data. Red values indicate species were positively correlated with clinical data, while blue ones indicate the species were negatively correlated with clinical data. Significant associations (*P*<0.05) are indicated by asterisks, with * represents *P*<0.05, ** represents *P*<0.01 and *** represents *P*<0.001. WBC, white blood cell; NE, neutrophil; CRP, C-reactive protein; PCT, procalcitonin; IL-6, interleukin-6; ESR, erythrocyte sedimentation rate; D-D, D-Dimer; BNP, brain natriuretic peptide; K, potassium ions; Na, sodium ion; Cl, chloride ion; Ca, calcium ion; LDH, lactic dehydrogenase; CK, creatine kinase; MYO, Myoglobin; CTnl, cardiac troponin I; ALT, alanine transaminase; AST, aspartate transferase; BUN, blood urea nitrogen; CREA, creatinine.

As a respiratory organ, the lungs perform important gas exchange functions and also act as a vehicle to maintain the dynamic balance of microbial communities between the organism and the external environment, as well as between the upper and lower airways. A series of previous studies have shown that imbalance of microbiota in the lung may be closely associated with the development of pulmonary (infectious) diseases. Therefore, while we clarified the characteristics of the responsible pathogen spectrum in different subgroups of *Chlamydia* infection, we also wished to further investigate the effects of *Chlamydia* infection on the microbiota of the lower respiratory tract by analyzing the characteristics of the lung microbial communities detected by mNGS and their differences between different subgroups of *Chlamydia* infection. **Figure 3** shows the distribution status (species level) of microbial communities in different *Chlamydia* infection subgroups and clinical controls, and provides overall information on the lung microbiota in patients with and without chlamydial infections. Using mNGS, we detected a total of 466 microbial species in all samples, most of which were bacteria (n=436), with a small number of fungi (n=23) and viruses (n=7). **Figure 3A** serves as a heatmap of the relative abundance of microbial species, showing the top 70 bacteria and all fungi and viruses in the 43 samples involved in this study.

To further investigate the effect of *Chlamydia* infection on the lung microbiota, we performed a comparative analysis of the microbial communities of 40 BALF samples from different *Chlamydia* infection subgroups (CP and PA groups) and clinical control groups (O and N groups). Compared to the control group N, the diversity of fungal and viral species was significantly reduced in the *Chlamydia*-infected groups (CP and PA groups) (**Figure 3B**; **Supplementary Table 3**). Additionally, the dominant microbial species in different chlamydial infection subgroups were not consistent, with the enriched species in the CP group being *Acinetobacter nosocomialis* (8.6%), *Aci. seifertii* (8.5%) and *Aci. lwoffii* (8.1%), whereas the superior species in the PA group were *Abiotrophia defectiva* (13.0%), *A. para-adiacens* (10.9%) and *Aci. seifertii* (11.0%).

Based on the classical Alpha-diversity Shannon and Simpson index analysis shown in **Figure 3C**, there was no statistical difference of the lung microbial community detected between the CP and N groups. However, the lung microbial community diversity of the PA group (*P* = 0.0039 and *P* = 0.0039) and the O group (*P* = 0.033 and *P* = 0.037) (Shannon and Simpson) were both significantly lower than that of the N group. Notably, the PA group had the lowest level of Alpha-diversity in the lung microbial community, suggesting that co-infection with *C. psittaci* and *C. abortus* might have important effects on the composition and diversity of the lung microbiome.

In addition, with two parameters of relative abundance and frequency, we performed a collaborative analysis of the top 50 microorganisms (including the top 30 bacteria, top 15 fungi, and top 5 viruses) to identify the specific microorganisms in the different clinical subgroups (**Figure 3D**). The results showed that the overall relative abundance and frequencies of microorganisms in the *Chlamydia*-infected group were lower than those in the N and O groups. Also, we identified some microbial species that were specifically enriched in different chlamydial infection subgroups and clinical control groups, such as *Actinomyces timonensis* (*P* = 2.7e-04), *Act. johnsonii* (*P* = 3.8e-02) and *Aci. johnsonii* (*P* = 3.5e-02) in the O group, *Aci. lwoffii* (*P* = 1.3e-02) and *Act. mediterranea* (*P* = 2.1e-02) were enriched in group N, *Act. oris* (*P* = 3.7e-02) in group PA, and *Aci. pittii* (*P* = 4.7e-02) in both groups PA and N.

### Correlation analysis between lung microbiome and clinical indices of *Chlamydia-*infection patients

We further wanted to understand whether the effect of *Chlamydia* infection on the microbiota diversity of the patient’s lower respiratory tract correlated with clinical status. After mentioned-above screening for microorganisms specifically enriched in each clinical subgroup, we performed a correlation analysis based on the spearman test using the relative abundance of these differential microorganisms and the infection-related clinical parameters of the corresponding patients (**Figure 3E**). The results showed that *Act. timonensis*, which was enriched in group O, was significantly and positively correlated with WBC (R = 0.36, *P* = 0.033) and negatively correlated with calcium ions (Ca^2+^) (R = -0.48, *P* = 0.044). *Aci. lwoffii* that enriched in group N was significantly negatively correlated with WBC (R = -0.41, *P* = 0.014), NE (R = -0.50, *P* = 0.002) and K^+^ (R = -0.53, *P* = 0.005). *Act. mediterranea* enriched in group N was significantly positively correlated with WBC (R = 0.37, *P* = 0.024) and creatinine (CREA) (R = 0.52, *P* = 0.022). *Aci. johnsonii* enriched in the O group showed a significantly positive correlation with K^+^ (R = 0.40, *P* = 0.036) and a significantly negative correlation with creatine kinase (CK) (R = -0.64, *P* = 0.008). *Act. oris* enriched in the PA group showed a significantly negative correlation with Ca^2+^ (R = -0.48, *P* = 0.044). *Aci. pittii* enriched in PA and N groups showed a significantly positive correlation with WBC (R = 0.39, *P* = 0.018) and a significantly negative correlation with LDH (R = -0.49, *P* = 0.023). These findings suggested that the dynamics of the pulmonary microbiota were closely related to the clinical parameters of patients after *Chlamydia* infection.

In order to understand the impact of chlamydial pneumonia-related pathogens on the composition and diversity of the patient’s lung microbiota more precisely, we thus performed a spearman test-based correlation analysis of the three representative responsible pathogens in the chlamydial infection group and the key commensal microorganisms specifically enriched in different clinical subgroups described above (**Supplementary Figure 3A**). It was interestingly to note that the abundance of *C. psittaci* were significantly and negatively correlated with that of *Act. mediterranea* (R= - 0.34, *P* = 0.033; **Supplementary Figures 3A, B**), *Aci. johnsonii* (R = -0.43, *P* = 0.006; **Supplementary Figures 3A, C**), *Act. oris* (R = -0.32, *P* = 0.044; **Supplementary Figures 3A, D**) and *Act. johnsonii* (R = -0.46, *P* = 0.003; **Supplementary Figures 3A, E**). Given these facts, we hypothesized that after infection, *C. psittaci* might alter the composition and diversity of the pulmonary microbiota by decreasing the relative abundance of certain pulmonary commensal microorganisms, which in turn directly affect the clinical status of the patient and the course of pulmonary infection.

## Discussion

In this study, we performed a comprehensive statistical analysis of the clinical characteristics of patients with suspected chlamydial pneumonia and classified these patients into different clinical subgroups. Pneumonia caused by *C. psittaci* infection is often misdiagnosed due to atypical clinical features and other diagnostic challenges. (Tang et al., 2022; Yang et al., 2022). Our study also found that most patients with suspected chlamydial pneumonia had no history of exposure to poultry, pets, or other farm animals, and they all had non-specific manifestations of pulmonary infection, such as fever, cough, and expectoration. These problems somehow pose a great challenge for rapid clinical confirmation of chlamydial infection. Previous studies have found that patients with pulmonary infection with *Chlamydia* have significantly elevated levels of inflammation-related markers in their peripheral blood, followed by a dramatic decrease to near normal levels after effective drug treatment (Li et al., 2021b). In our study, patients from different subgroups showed significant differences in clinical symptoms, peripheral blood inflammatory indicators and electrolyte levels. Specifically, patients with co-infection of *C. psittaci* and *C. abortus* had the highest peripheral blood CRP levels compared to other *Chlamydia* infection subgroups, indicating that co-infection might lead to severer pulmonary infections and a pan-systemic inflammatory state (Zhang et al., 2022b; Calderwood et al., 2023). Thus, the analysis of the responsible pathogen profile and the lung microbiota characteristics performed in this study for different patterns of *Chlamydia* infection and their correlation with the clinical status of the patients seems to be essential.

While the genomes of *C. psittaci* and *C. abortus* are highly homologous (Stephens et al., 2009; Wheelhouse and Longbottom, 2012), according to the **Supplementary Table 1**, the three infection groups, *C. psittaci* mono-infection, *C. abortus* mono-infection and mixed *C. psittaci* and *C. abortus* infections, did clearly showed unique spectrum of causative pathogens, which makes the etiological identification of *Chlamydia* pneumonia more complex and brings a great challenge to the pathogenic diagnosis in patients with *Chlamydia* pneumonia. Although there was only one BALF sample included in the CA group, it still showed a primary tendency. Of course, for a more solid conclusion, further researches are needed in the future. In recent years, as a high-throughput sequencing-based assay, mNGS has demonstrated good diagnostic performance for clinical infection pathogens, i.e., it can identify different types of pathogens, such as bacteria, fungi, viruses and parasites, in a single run, which is valuable for rapid and accurate diagnosis of mixed infections (Liang et al., 2022). However, previous studies have shown that there are some limitations to mNGS’s use in the detection of virus, e.g. mNGS may not be able to detect low-level viral infections due to insufficient sequencing depth and coverage to identify low-abundance viral reads within a complex sample, mNGS may be prone to contamination, leading to the detection of false-positive viral sequences, mNGS may have difficulty detecting viruses with highly variable genomes due to bad alignment with the reference genomes, human genes in clinical samples also greatly influence the sensitivity of mNGS to detect viral pathogens. Therefore, all of the above shortcomings in viral detection raise concerns about the ability of mNGS to effectively detect low-abundance rare and unusual pathogens in clinical samples (Deng et al., 2020; Wang et al., 2020; Gu et al., 2021; Li et al., 2022). Our results showed that mNGS can effectively distinguish different species of *Chlamydia* from clinical samples and identify co-infections with other pathogenic microorganisms, such as *C. tropicalis*, *S. pneumoniae*, *H. influenzae*, *Enterococcus faecalis* and EBV. Furthermore, although due to the limited amounts of samples included in the CA group, it seemed difficult to statistically compare the pathogenic abundance between CA and CP groups. However, for the BALF sample of CA (i.e. CA1), there were five causative pathogens identified, which was much less than that in the CP group (10 in total), according to **Supplementary Table 1**. Moreover, based on clinical observations, patients in the CP group did show severer symptoms than that in the CA group. Therefore, we draw a primary conclusion here that there are more co-infecting pathogens identified in patients infected with *C. psittaci* compared to those infected with *C. abortus*, suggesting that *C. psittaci* infection might cause a potentially higher risk of co-infection, which in turn leads to severer clinical symptoms and a longer disease course, and this observation was also in accordance with the results of previous study (Liang et al., 2022). Interestingly, despite having a lower risk of co-infection, we observed unique *C. albicans* and *S. pneumoniae* co-infections in *C. abortus-*infected patients. Previous studies have found that *C. albicans* and *S. pneumoniae* co-infection was frequently observed in cases of acute mixed polymicrobial infections, and its presence may lead to worsening clinical symptoms (Eichelberger and Cassat, 2021). Although *C. abortus*-caused severe symptoms of infection have not been detected in this study, clinicians should still pay more attention on cases of mixed infections involving *C. abortus* and try to avoid the development of severe symptoms.

The microbiota of the lower respiratory tract contains specific ecological niches of commensal and pathogenic microorganisms, which play an important role in determining the onset and progression of disease (Man et al., 2017; Xia et al., 2022). The homeostatic state of microecological diversity in the lung may be influenced by various biotic or abiotic conditions (Liu et al., 2022a). Clinical studies based on infectious pneumonia have shown that increased colonization by opportunistic pathogens may lead to imbalance in the lower respiratory microecology and trigger symptoms of infection in the lower respiratory tract (De Pascale et al., 2021; Narendrakumar and Ray, 2022). Changes in the microbiota have been reported during lower respiratory tract infections and such changes are strongly associated with the course or prognosis of pneumonia (Hanada et al., 2018). Thus, it is evident that a clearer elucidation of the compositional changes in the lower respiratory microbiome would help to understand the pathogenic process and mechanisms of infectious pneumonia. A previous series of studies revealed the pulmonary microbial compositions by non-targeted pathogen metagenomics or 16S rRNA sequencing, that the microbiomes of children with bacterial meningitis (Liao et al., 2021), patients with refractory *Mycoplasma pneumoniae* pneumonia (Zhou et al., 2022), patients with tuberculosis (Ding et al., 2021; Xiao et al., 2022), and patients with invasive pulmonary aspergillosis (Hérivaux et al., 2022). Unlike mNGS that can support high-resolution species-level microbial identification, 16S rRNA sequencing can only achieve precise identification and annotation at the bacterial genus level, which can neither distinguish between microbial species of the same genus with very high homology nor provide comprehensive microbial information (bacteria, fungi, viruses, *Mycoplasma*/*Chlamydia*, etc.) (Man et al., 2019). Therefore, in this study, we investigated the characteristics of the pulmonary microbiota of patients with and without *Chlamydia* infection by mNGS. BALF samples are widely used for pathogen detection in infectious diseases of the lower respiratory tract because they carry less oral microbial contamination and can yield accurate and representative lung microbial information (Bingula et al., 2020; Fenn et al., 2022; Jin et al., 2022; Zhang et al., 2022a). In this study, we characterized the lung microbiota using 40 BALF samples from patients with different *Chlamydia* infections and non-*Chlamydia* infections. The results showed that the lower respiratory microecological diversity was significantly lower in patients with *Chlamydia* infection than those with non-*Chlamydia* infection, similar to previous observations in patients with tuberculosis or ventilator-associated pneumonia (Hu et al., 2020; Fenn et al., 2022). Proximally, patients with invasive pulmonary aspergillosis also experienced reduced pulmonary microecological diversity, in particular, a significant reduction in the relative abundance of bacterial genera (Hérivaux et al., 2022). In our study, a significant reduction in the relative abundance of some bacterial species, such as *Aci. lwoffii* and *Act. mediterranea*, was also observed in patients with *Chlamydia* infection. Despite a series of clinical case reports showing that *Aci. lwoffii* can cause peritonitis, liver abscesses and even severe pneumonia (Toyoshima et al., 2010; Singh et al., 2016; Dammak et al., 2022), studies have shown that *Aci. lwoffii* has positive effects. For example, *Aci. lwoffii* can participate in the activation of macrophages in the lung, regulating the shift of M2a and M2c macrophages to M2b macrophages, thereby inhibiting the type 2 response that causes asthma (Kang et al., 2022). On the other hand, although no direct association of *Act. mediterranea* with human disease has been reported, *Actinomyces spp.* have been implicated in different processes of human physiology and pathology. For example, a study based on sequencing of the 16S ribosomal RNA gene V3-V4 region found that, compared to COVID-19 patients with common type, the abundance of commersal bacteria such as *Actinomycetes* was significantly lower in samples from patients with severe disease (Chen et al., 2022). Considering the pathogenic mechanisms, the reduction in microecological diversity may lead to a significant colonization of the lower respiratory tract by the dominant pathogens (Natalini et al., 2022), which in turn may have a negative impact on the infection process. Furthermore, our study found that although reduced pulmonary microecological diversity occurred in both *Chlamydia*-infected and non-*Chlamydia*-infected patients, the microbial species with altered relative abundance in the lower respiratory tract were significantly different in these two patient groups, suggesting that *Chlamydia* infection shapes the characteristics of the pulmonary microbiota in unique disease states.

Although there are few studies on the correlation between clinical parameters and microbiota characteristics in patients with pulmonary infections, we can still find some clues, for example, changes in various clinical indicators in patients with *Pneumocystis* pneumonia are closely associated with changes in the relative abundance of their low respiratory tract microbiota (Zhu et al., 2022b). In the present study, we observed significant differences between patients with and without *Chlamydia* infection in terms of clinical characteristics, key responsible pathogens and representative lower respiratory tract commensal microorganisms. Therefore, we attempted to correlate the above differential data at different levels to further explore the possible mechanisms by which *Chlamydia* and its commensal microorganisms influence the course of pulmonary infections in patients. We found that infection with *C. psittaci* significantly decreased the relative abundance of commensal microorganisms in the lower respiratory tract, such as *Actinomyces mediterraneae*, *Actinomyces johnsonii, Actinomyces ori, Actinomyces timonensis* and *Acinetobacter johnsonii*, and that changes in the relative abundance of these microorganisms in turn showed significant correlations with changes in clinical indicators related to infection or inflammation, for example, *Actinomyces timonensis* was positively correlated with white blood cell count, but negatively correlated with Ca^+^, while *Actinomyces mediterraneae* positively correlated with both white blood cell count and CREA.

On the other hand, an increase in the relative abundance of *Chlamydia* was accompanied by a significant increase in the levels of white blood cell count, CRP, and K^+^, while levels of Na^+^ decreased. Thus, the present study provides, to some extent, possible evidence supporting a close correlation among *Chlamydia* infection, altered microecological diversity of the patient’s lungs and clinical parameters related to infection or inflammation, which also provides a possible research direction for unveiling the pathogenic mechanisms of pulmonary infections caused by *Chlamydia*.

Indeed, we are aware of the limitations of the present study. First, this study included a limited population of patients with chlamydial pneumonia, especially in the single infection group of *C. abortus*. Because of this, we were unable to obtain sufficient BALF samples to explore the characteristics of the pulmonary microbiome of patients with *C. abortus* infection. Therefore, increased amounts of clinical samples might be required in future studies to draw more accurate conclusions about the microecological diversity of the lower respiratory tract and its clinical relevance in patients with chlamydial pneumonia. Second, although we found that *Chlamydia* infection can remodel the microbiome of the lower respiratory tract, it remains unclear what changes occur in the pulmonary microbiome and their clinical relevance during treatment and even after cure in patients with *Chlamydia* pneumonia. Furthermore, some studies have reported that microbial imbalance can induce systemic metabolic alterations (Devaraj et al., 2013; Nieuwdorp et al., 2014) and host immune responses (Xia et al., 2022). Meanwhile, microorganisms such as *Bacillus* spp., *Actinomyces* spp. and even *Chlamydia psittaci* or *Chlamydia abortus* can directly or indirectly regulate the expression of host pro-inflammatory factors or important disease-related genes such as METTLE3, thus affecting the physiological and pathological processes of the host (Pan et al., 2017; Chen et al., 2020a; Gómez-García et al., 2022; Wang et al., 2022; Xu et al., 2022; Zhang et al., 2022b). These previous studies will help us to conduct future research on the direction of microbial-host immune interactions in terms of how microbes and their metabolites regulate pro-inflammatory signaling pathways, and how microbes and their metabolites are involved in the regulation of signaling pathways and expression of key targets related to important diseases such as inflammation or tumours.

In summary, we detected and analyzed in this study the responsible pathogen profile, infection patterns, lung microbiota characteristics and their correlation with the clinical characteristics of patients with and without *Chlamydia* infection by mNGS. Our results suggest that *Chlamydia* infection disrupts the dynamic balance of the pulmonary microbiome, which may impact disease severity. The effects of *Chlamydia* infection on the clinical symptoms and the course of the disease in patients warrant further studies. Our findings help to deepen our understanding of the pathogenesis of chlamydial pneumonia, especially mixed *Chlamydia* infections.

## Data availability statement

The datasets presented in this study can be found in online repositories. The names of the repository/repositories and accession number(s) can be found below: NCBI, SRA: PRJNA924534.

## Ethics statement

The studies involving human participants were reviewed and approved by The Ethics Commitee of the Hunan Provincial People’s Hospital, The First Affiliated Hospital of Hunan Normal University. The patients/participants provided their written informed consent to participate in this study.

## Author contributions

GX, QH and YL are the primary physicians who provided diagnosis and treatment of the patients. GX, QH, XC, WW, LW and RR collected and analyzed clinical and sequencing data. PD, WG and OW prepared some paragraphs of the manuscript in Chinese. GX, QH, XC, WW, RR and YL wrote the manuscript. All authors contributed to the article and approved the submitted version.

## Supplementary material

Supplementary Figure 1 Receiver operating characteristic (ROC) curve of mNGS test for *Chlamydia abortus* **(A)** and *Chlamydia psittaci* **(B)** when clinical diagnosis was regarded as the gold standard.

Supplementary Figure 2 Spearman’s test showed that *Chlamydia psittaci* was significantly positively correlated with WBC **(A)**, CRP **(B)**, K^+^ **(C)** and significantly negatively correlated with Na^+^ **(D)**, and *Chlamydia abortus* was was significantly positively correlated with WBC **(E)**, CRP **(F)**, K^+^ **(G)** and negatively correlated with Na^+^ **(H)**.

Supplementary Figure 3 Spearman correlation analysis between responsible pathogens with microbial species showed that *Chlamydia psittaci* was negatively correlated with micrbiobial species of *Actinomyces mediterranea* **(A, B)**, *Acinetobacter johnsonii* **(A, C)**, *Actinomyces oris* **(A, D)** and *Actinomyces johnsonii* **(A, E)**.

## References

[B1] Barati S. Moori-Bakhtiari N. Najafabadi M. G. Momtaz H. Shokuhizadeh L. (2017). The role of zoonotic chlamydial agents in ruminants abortion. Iran. J. Microbiol. 9, 288–294. 29296274 PMC5748448

[B2] Bingula R. Filaire E. Molnar I. Delmas E. Berthon J.-Y. Vasson M.-P.. (2020). Characterisation of microbiota in saliva, bronchoalveolar lavage fluid, non-malignant, peritumoural and tumour tissue in non-small cell lung cancer patients: a cross-sectional clinical trial. Respir. Res. 21, 129. doi: 10.1186/s12931-020-01392-2 32450847 PMC7249392

[B3] Calderwood C. J. Reeve B. W. Mann T. Palmer Z. Nyawo G. Mishra H.. (2023). Clinical utility of c-reactive protein-based triage for presumptive pulmonary tuberculosis in south African adults. J. Infect. 86, 24–32. doi: 10.1016/j.jinf.2022.10.041 36375640 PMC10567578

[B4] Chen X. Cao K. Wei Y. Qian Y. Liang J. Dong D.. (2020b). Metagenomic next-generation sequencing in the diagnosis of severe pneumonias caused by chlamydia psittaci. Infection 48, 535–542. doi: 10.1007/s15010-020-01429-0 32314307 PMC7223968

[B5] Chen Q. Li Y. Yan X. Sun Z. Wang C. Liu S.. (2020a). Chlamydia psittaci plasmid-encoded CPSIT_P7 elicits inflammatory response in human monocytes *via* TLR4/Mal/MyD88/NF-κB signaling pathway. Front. Microbiol. 11. doi: 10.3389/fmicb.2020.578009 PMC7744487 33343522

[B6] Chen J. Liu X. Liu W. Yang C. Jia R. Ke Y.. (2022). Comparison of the respiratory tract microbiome in hospitalized COVID-19 patients with different disease severity. J. Med. Virol. 94, 5284–5293. doi: 10.1002/jmv.28002 35838111 PMC9349541

[B7] Cheong H. C. Lee C. Y. Q. Cheok Y. Y. Tan G. M. Y. Looi C. Y. Wong W. F. (2019). Chlamydiaceae: diseases in primary hosts and zoonosis. Microorganisms 7, 146. doi: 10.3390/microorganisms7050146 31137741 PMC6560403

[B8] Corsaro D. Greub G. (2006). Pathogenic potential of novel chlamydiae and diagnostic approaches to infections due to these obligate intracellular bacteria. Clin. Microbiol. Rev. 19, 283–297. doi: 10.1128/CMR.19.2.283-297.2006 16614250 PMC1471994

[B9] Corsaro D. Venditti D. (2004). Emerging chlamydial infections. Crit. Rev. Microbiol. 30, 75–106. doi: 10.1080/10408410490435106 15239381

[B10] Dammak N. Chaker H. Agrebi I. Toumi S. Mseddi F. Kammoun K.. (2022). Acinetobacter lwoffi peritonitis in peritoneal dialysis: two cases report. Tunis. Med. 100, 481–484. 36206068 PMC9615012

[B11] Deng X. Achari A. Federman S. Yu G. Somasekar S. Bártolo I.. (2020). Metagenomic sequencing with spiked primer enrichment for viral diagnostics and genomic surveillance. Nat. Microbiol. 5, 443–454. doi: 10.1038/s41564-019-0637-9 31932713 PMC7047537

[B12] De Pascale G. De Maio F. Carelli S. De Angelis G. Cacaci M. Montini L.. (2021). Staphylococcus aureus ventilator-associated pneumonia in patients with COVID-19: clinical features and potential inference with lung dysbiosis. Crit. Care 25, 197. doi: 10.1186/s13054-021-03623-4 34099016 PMC8182737

[B13] Devaraj S. Hemarajata P. Versalovic J. (2013). The human gut microbiome and body metabolism: implications for obesity and diabetes. Clin. Chem. 59, 617–628. doi: 10.1373/clinchem.2012.187617 23401286 PMC3974587

[B14] Ding L. Liu Y. Wu X. Wu M. Luo X. Ouyang H.. (2021). Pathogen metagenomics reveals distinct lung microbiota signatures between bacteriologically confirmed and negative tuberculosis patients. Front. Cell. Infect. Microbiol. 11. doi: 10.3389/fcimb.2021.708827 PMC8475726 34589441

[B15] Duan Z. Gao Y. Liu B. Sun B. Li S. Wang C.. (2022). The application value of metagenomic and whole-genome capture next-generation sequencing in the diagnosis and epidemiological analysis of psittacosis. Front. Cell. Infect. Microbiol. 12. doi: 10.3389/fcimb.2022.872899 PMC9207344 35734579

[B16] Eichelberger K. R. Cassat J. E. (2021). Metabolic adaptations during staphylococcus aureus and candida albicans Co-infection. Front. Immunol. 12. doi: 10.3389/fimmu.2021.797550 PMC8692374 34956233

[B17] Fenn D. Abdel-Aziz M. I. van Oort P. M. P. Brinkman P. Ahmed W. M. Felton T.. (2022). Composition and diversity analysis of the lung microbiome in patients with suspected ventilator-associated pneumonia. Crit. Care 26, 203. doi: 10.1186/s13054-022-04068-z 35794610 PMC9261066

[B18] Gómez-García A. P. López-Vidal Y. Pinto-Cardoso S. Aguirre-García M. M. (2022). Overexpression of proinflammatory cytokines in dental pulp tissue and distinct bacterial microbiota in carious teeth of Mexican individuals. Front. Cell. Infect. Microbiol. 12. doi: 10.3389/fcimb.2022.958722 PMC9772992 36569197

[B19] Gu W. Deng X. Lee M. Sucu Y. D. Arevalo S. Stryke D.. (2021). Rapid pathogen detection by metagenomic next-generation sequencing of infected body fluids. Nat. Med. 27, 115–124. doi: 10.1038/s41591-020-1105-z 33169017 PMC9020267

[B20] Hanada S. Pirzadeh M. Carver K. Y. Deng J. C. (2018). Respiratory viral infection-induced microbiome alterations and secondary bacterial pneumonia. Front. Immunol. 9. doi: 10.3389/fimmu.2018.02640 PMC6250824 30505304

[B21] Harkinezhad T. Verminnen K. Van Droogenbroeck C. Vanrompay D. (2007). Chlamydophila psittaci genotype E/B transmission from African grey parrots to humans. J. Med. Microbiol. 56, 1097–1100. doi: 10.1099/jmm.0.47157-0 17644718

[B22] Hérivaux A. Willis J. R. Mercier T. Lagrou K. Gonçalves S. M. Gonçales R. A.. (2022). Lung microbiota predict invasive pulmonary aspergillosis and its outcome in immunocompromised patients. Thorax 77, 283–291. doi: 10.1136/thoraxjnl-2020-216179 34172558 PMC8867272

[B23] Hu Y. Cheng M. Liu B. Dong J. Sun L. Yang J.. (2020). Metagenomic analysis of the lung microbiome in pulmonary tuberculosis - a pilot study. Emerg. Microbes Infect. 9, 1444–1452. doi: 10.1080/22221751.2020.1783188 32552447 PMC7473061

[B24] Imkamp F. Albini S. Karbach M. Kimmich N. Spinelli C. Herren S.. (2022). Zoonotic chlamydiae as rare causes of severe pneumonia. Swiss Med. Wkly. 152, w30102. doi: 10.4414/SMW.2022.w30102 35019255

[B25] Jin X. Li J. Shao M. Lv X. Ji N. Zhu Y.. (2022). Improving suspected pulmonary infection diagnosis by bronchoalveolar lavage fluid metagenomic next-generation sequencing: a multicenter retrospective study. Microbiol. Spectr. 10, e02473–e02421. doi: 10.1128/spectrum.02473-21 PMC9431624 35943274

[B26] Joseph S. J. Marti H. Didelot X. Castillo-Ramirez S. Read T. D. Dean D. (2015). *Chlamydiaceae* genomics reveals interspecies admixture and the recent evolution of *Chlamydia abortus* infecting lower mammalian species and humans. Genome Biol. Evol. 7, 3070–3084. doi: 10.1093/gbe/evv201 26507799 PMC4994753

[B27] Kang H. Bang J.-Y. Mo Y. Shin J. W. Bae B. Cho S.-H.. (2022). Effect of acinetobacter lwoffii on the modulation of macrophage activation and asthmatic inflammation. Clin. Exp. Allergy J. Br. Soc. Allergy Clin. Immunol. 52, 518–529. doi: 10.1111/cea.14077 34874580

[B28] Li N. Cai Q. Miao Q. Song Z. Fang Y. Hu B. (2021a). High-throughput metagenomics for identification of pathogens in the clinical settings. Small Methods 5, 2000792. doi: 10.1002/smtd.202000792 33614906 PMC7883231

[B29] Li N. Li S. Tan W. Wang H. Xu H. Wang D. (2021b). Metagenomic next-generation sequencing in the family outbreak of psittacosis: the first reported family outbreak of psittacosis in China under COVID-19. Emerg. Microbes Infect. 10, 1418–1428. doi: 10.1080/22221751.2021.1948358 34176434 PMC8284143

[B30] Li S. Tong J. Liu Y. Shen W. Hu P. (2022). Targeted next generation sequencing is comparable with metagenomic next generation sequencing in adults with pneumonia for pathogenic microorganism detection. J. Infect. 85, e127–e129. doi: 10.1016/j.jinf.2022.08.022 36031154

[B31] Liang Y. Dong T. Li M. Zhang P. Wei X. Chen H.. (2022). Clinical diagnosis and etiology of patients with chlamydia psittaci pneumonia based on metagenomic next-generation sequencing. Front. Cell. Infect. Microbiol. 12. doi: 10.3389/fcimb.2022.1006117 PMC9606567 36310873

[B32] Liao H. Zhang Y. Guo W. Wang X. Wang H. Ye H.. (2021). Characterization of the blood and cerebrospinal fluid microbiome in children with bacterial meningitis and its potential correlation with inflammation. mSystems 6, e00049–e00021. doi: 10.1128/mSystems.00049-21 34100633 PMC8269202

[B33] Liu M. Wen Y. Ding H. Zeng H. (2022b). Septic shock with chlamydia abortus infection. Lancet Infect. Dis. 22, 912. doi: 10.1016/S1473-3099(21)00756-8 35643111

[B34] Liu C. Wu K. Sun T. Chen B. Yi Y. Ren R.. (2022a). Effect of invasive mechanical ventilation on the diversity of the pulmonary microbiota. Crit. Care 26, 252. doi: 10.1186/s13054-022-04126-6 35996150 PMC9394019

[B35] Man W. H. de Steenhuijsen Piters W. A. A. Bogaert D. (2017). The microbiota of the respiratory tract: gatekeeper to respiratory health. Nat. Rev. Microbiol. 15, 259–270. doi: 10.1038/nrmicro.2017.14 28316330 PMC7097736

[B36] Man W. H. van Houten M. A. Mérelle M. E. Vlieger A. M. Chu M. L. J. N. Jansen N. J. G.. (2019). Bacterial and viral respiratory tract microbiota and host characteristics in children with lower respiratory tract infections: a matched case-control study. Lancet Respir. Med. 7, 417–426. doi: 10.1016/S2213-2600(18)30449-1 30885620 PMC7172745

[B37] Marti H. Jelocnik M. (2022). Animal chlamydiae: a concern for human and veterinary medicine. Pathogens 11, 364. doi: 10.3390/pathogens11030364 35335688 PMC8951289

[B38] Miao Q. Ma Y. Wang Q. Pan J. Zhang Y. Jin W.. (2018). Microbiological diagnostic performance of metagenomic next-generation sequencing when applied to clinical practice. Clin. Infect. Dis. 67, S231–S240. doi: 10.1093/cid/ciy693 30423048

[B39] Narendrakumar L. Ray A. (2022). “Respiratory tract microbiome and pneumonia,” in Progress in molecular biology and translational science (Elsevier), 97–124. doi: 10.1016/bs.pmbts.2022.07.002 36280326

[B40] Natalini J. G. Singh S. Segal L. N. (2022). The dynamic lung microbiome in health and disease. Nat. Rev. Microbiol. 21, 222–235 doi: 10.1038/s41579-022-00821-x PMC9668228 36385637

[B41] Nieuwdorp M. Gilijamse P. W. Pai N. Kaplan L. M. (2014). Role of the microbiome in energy regulation and metabolism. Gastroenterology 146, 1525–1533. doi: 10.1053/j.gastro.2014.02.008 24560870

[B42] Ortega N. Caro M. R. Gallego M. C. Murcia-Belmonte A. Álvarez D. del Río L.. (2015). Isolation of chlamydia abortus from a laboratory worker diagnosed with atypical pneumonia. Ir. Vet. J. 69, 8. doi: 10.1186/s13620-016-0067-4 27446530 PMC4955219

[B43] Pan Q. Zhang Q. Chu J. Pais R. Liu S. He C.. (2017). Chlamydia abortus Pmp18.1 induces IL-1β secretion by TLR4 activation through the MyD88, NF-κB, and caspase-1 signaling pathways. Front. Cell. Infect. Microbiol. 7. doi: 10.3389/fcimb.2017.00514 PMC5741698 29326885

[B44] Pospischil A. Thoma R. Hilbe M. Grest P. Zimmermann D. Gebbers J.-O. (2002). Abort beim menschen durch chlamydophila abortus (Chlamydia psittaci serovar 1). Schweiz. Arch. Für Tierheilkd. 144, 463–466. doi: 10.1024/0036-7281.144.9.463 12677684

[B45] Qin X.-C. Huang J. Yang Z. Sun X. Wang W. Gong E.. (2022). Severe community-acquired pneumonia caused by *Chlamydia psittaci* genotype E/B strain circulating among geese in lishui city, zhejiang province, China. Emerg. Microbes Infect. 11, 2715–2723. doi: 10.1080/22221751.2022.2140606 36287125 PMC9661978

[B46] Sachse K. Bavoil P. M. Kaltenboeck B. Stephens R. S. Kuo C.-C. Rosselló-Móra R.. (2015). Emendation of the family chlamydiaceae: proposal of a single genus, chlamydia, to include all currently recognized species. Syst. Appl. Microbiol. 38, 99–103. doi: 10.1016/j.syapm.2014.12.004 25618261

[B47] Shaw K. A. Szablewski C. M. Kellner S. Kornegay L. Bair P. Brennan S.. (2019). Psittacosis outbreak among workers at chicken slaughter plants, Virginia and Georgia, USA 2018. Emerg. Infect. Dis. 25, 2143–2145. doi: 10.3201/eid2511.190703 31625859 PMC6810211

[B48] Singh N. P. Sagar T. Nirmal K. Kaur I. R. (2016). Pyogenic liver abscess caused by acinetobacter lwoffii: a case report. J. Clin. Diagn. Res. JCDR 10, DD01–DD02. doi: 10.7860/JCDR/2016/18256.7943 PMC4963646 27504286

[B49] Stephens R. S. Myers G. Eppinger M. Bavoil P. M. (2009). Divergence without difference: phylogenetics and taxonomy of *Chlamydia* resolved. FEMS Immunol. Med. Microbiol. 55, 115–119. doi: 10.1111/j.1574-695X.2008.00516.x 19281563

[B50] Tang J. Tan W. Luo L. Xu H. Li N. (2022). Application of metagenomic next-generation sequencing in the diagnosis of pneumonia caused by chlamydia psittaci. Microbiol. Spectr. 10, e02384–e02321. doi: 10.1128/spectrum.02384-21 PMC9431268 35938720

[B51] Toyoshima M. Chida K. Suda T. (2010). Fulminant community-acquired pneumonia probably caused by acinetobacter lwoffii. Respirol. Carlton Vic 15, 867–868. doi: 10.1111/j.1440-1843.2010.01780.x 20546186

[B52] Wallensten A. Fredlund H. Runehagen A. (2014). Multiple human-to-human transmission from a severe case of psittacosis, Sweden, January–February 2013. Euro Surveill. 19(42): 20937. doi: 10.2807/1560-7917.ES2014.19.42.20937 25358043

[B53] Wang M. Li M. Ren R. Li L. Chen E.-Q. Li W.. (2020). International expansion of a novel SARS-CoV-2 mutant. J. Virol. 94, e00567–e00520. doi: 10.1128/JVI.00567-20 32269121 PMC7307084

[B54] Wang C. Li A. Shi Q. Yu Z. (2021). Metagenomic next-generation sequencing clinches diagnosis of leishmaniasis. Lancet 397, 1213. doi: 10.1016/S0140-6736(21)00352-4 33773632

[B55] Wang H. Wang Q. Chen J. Chen C. (2022). Association among the gut microbiome, the serum metabolomic profile and RNA m6A methylation in sepsis-associated encephalopathy. Front. Genet. 13. doi: 10.3389/fgene.2022.859727 PMC9006166 35432460

[B56] Wheelhouse N. Longbottom D. (2012). Endemic and emerging chlamydial infections of animals and their zoonotic implications: emerging chlamydial infections. Transbound Emerg. Dis. 59, 283–291. doi: 10.1111/j.1865-1682.2011.01274.x 22099945

[B57] Wu H. Feng L. Fang S. (2021). Application of metagenomic next-generation sequencing in the diagnosis of severe pneumonia caused by chlamydia psittaci. BMC Pulm. Med. 21, 300. doi: 10.1186/s12890-021-01673-6 34556069 PMC8461849

[B58] Xia X. Chen J. Cheng Y. Chen F. Lu H. Liu J.. (2022). Comparative analysis of the lung microbiota in patients with respiratory infections, tuberculosis, and lung cancer: a preliminary study. Front. Cell. Infect. Microbiol. 12. doi: 10.3389/fcimb.2022.1024867 PMC9663837 36389135

[B59] Xiao G. Cai Z. Guo Q. Ye T. Tang Y. Guan P.. (2022). Insights into the unique lung microbiota profile of pulmonary tuberculosis patients using metagenomic next-generation sequencing. Microbiol. Spectr. 10, e01901–e01921. doi: 10.1128/spectrum.01901-21 35196800 PMC8865484

[B60] Xu L. Zhao Z. Mai H. Tan X. Du Y. Fang C. (2022). Clinical and chest computed tomography features associated with severe chlamydia psittaci pneumonia diagnosed by metagenomic next-generation sequencing: a multicenter, retrospective, observational study. Med. (Baltimore) 101, e32117. doi: 10.1097/MD.0000000000032117 PMC9771292 36550834

[B61] Yang M. Yang D.-H. Yang H. Ding S.-Z. Liu C.-H. Yin H.-M.. (2022). Clinical characteristics of chlamydia psittaci pneumonia infection in central south China. Infect. Dis. Ther. doi: 10.1007/s40121-022-00662-4 PMC9207437 35723864

[B62] Yin Q. Li Y. Pan H. Hui T. Yu Z. Wu H.. (2022). Atypical pneumonia caused by chlamydia psittaci during the COVID-19 pandemic. Int. J. Infect. Dis. 122, 622–627. doi: 10.1016/j.ijid.2022.07.027 35842216 PMC9276535

[B63] Zaręba-Marchewka K. Szymańska-Czerwińska M. Niemczuk K. (2020). Chlamydiae – what’s new? J. Vet. Res. 64, 461–467. doi: 10.2478/jvetres-2020-0077 33367133 PMC7734683

[B64] Zhang J. Gao L. Zhu C. Jin J. Song C. Dong H.. (2022a). Clinical value of metagenomic next-generation sequencing by illumina and nanopore for the detection of pathogens in bronchoalveolar lavage fluid in suspected community-acquired pneumonia patients. Front. Cell. Infect. Microbiol. 12. doi: 10.3389/fcimb.2022.1021320 PMC9551279 36237436

[B65] Zhang Z. Wang P. Ma C. Wang J. Li W. Quan C.. (2022b). Host inflammatory response is the major factor in the progression of chlamydia psittaci pneumonia. Front. Immunol. 13. doi: 10.3389/fimmu.2022.929213 PMC9478202 36119044

[B66] Zhang Z. Zhou H. Cao H. Ji J. Zhang R. Li W.. (2022c). Human-to-human transmission of chlamydia psittaci in China 2020: an epidemiological and aetiological investigation. Lancet Microbe 3, e512–e520. doi: 10.1016/S2666-5247(22)00064-7 35617977

[B67] Zhou W. Chen J. Xi Z. Shi Y. Wang L. Lu A. (2022). Characteristics of lung microbiota in children’s refractory mycoplasma pneumoniae pneumonia coinfected with human adenovirus b. Can. J. Infect. Dis. Med. Microbiol. 2022, 1–8. doi: 10.1155/2022/7065890 PMC8786547 35082959

[B68] Zhu M. Liu S. Zhao C. Shi J. Li C. Ling S.. (2022b). Alterations in the gut microbiota of AIDS patients with pneumocystis pneumonia and correlations with the lung microbiota. Front. Cell. Infect. Microbiol. 12. doi: 10.3389/fcimb.2022.1033427 PMC9634167 36339339

[B69] Zhu C. Lv M. Huang J. Zhang C. Xie L. Gao T.. (2022a). Bloodstream infection and pneumonia caused by chlamydia abortus infection in China: a case report. BMC Infect. Dis. 22, 181. doi: 10.1186/s12879-022-07158-z 35197012 PMC8867867

