# Supplementary figures and images for: Clinical identification and microbiota analysis of Chlamydia psittaci- and Chlamydia abortus- pneumonia by metagenomic next-generation sequencing
Source: Front Cell Infect Microbiol. 2023 Jun 26;13:1157540. doi: 10.3389/fcimb.2023.1157540 (PMC10331293; doi:10.3389/fcimb.2023.1157540)

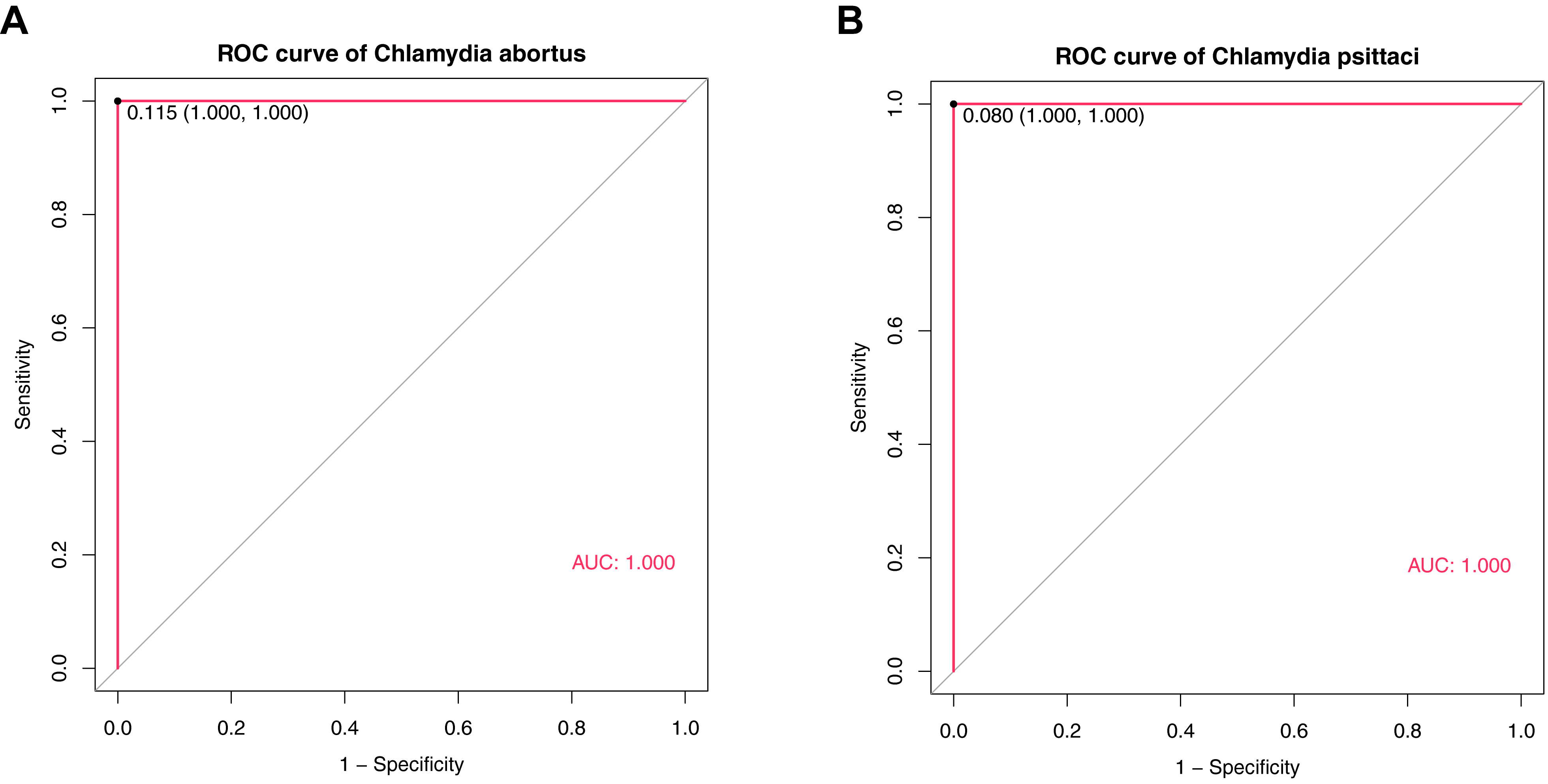

Supplement: Supplementary Figure 1 — Receiver operating characteristic (ROC) curve of mNGS test for Chlamydia abortus (A) and Chlamydia psittaci (B) when clinical diagnosis was regarded as the gold standard. [file Image_1.tif]

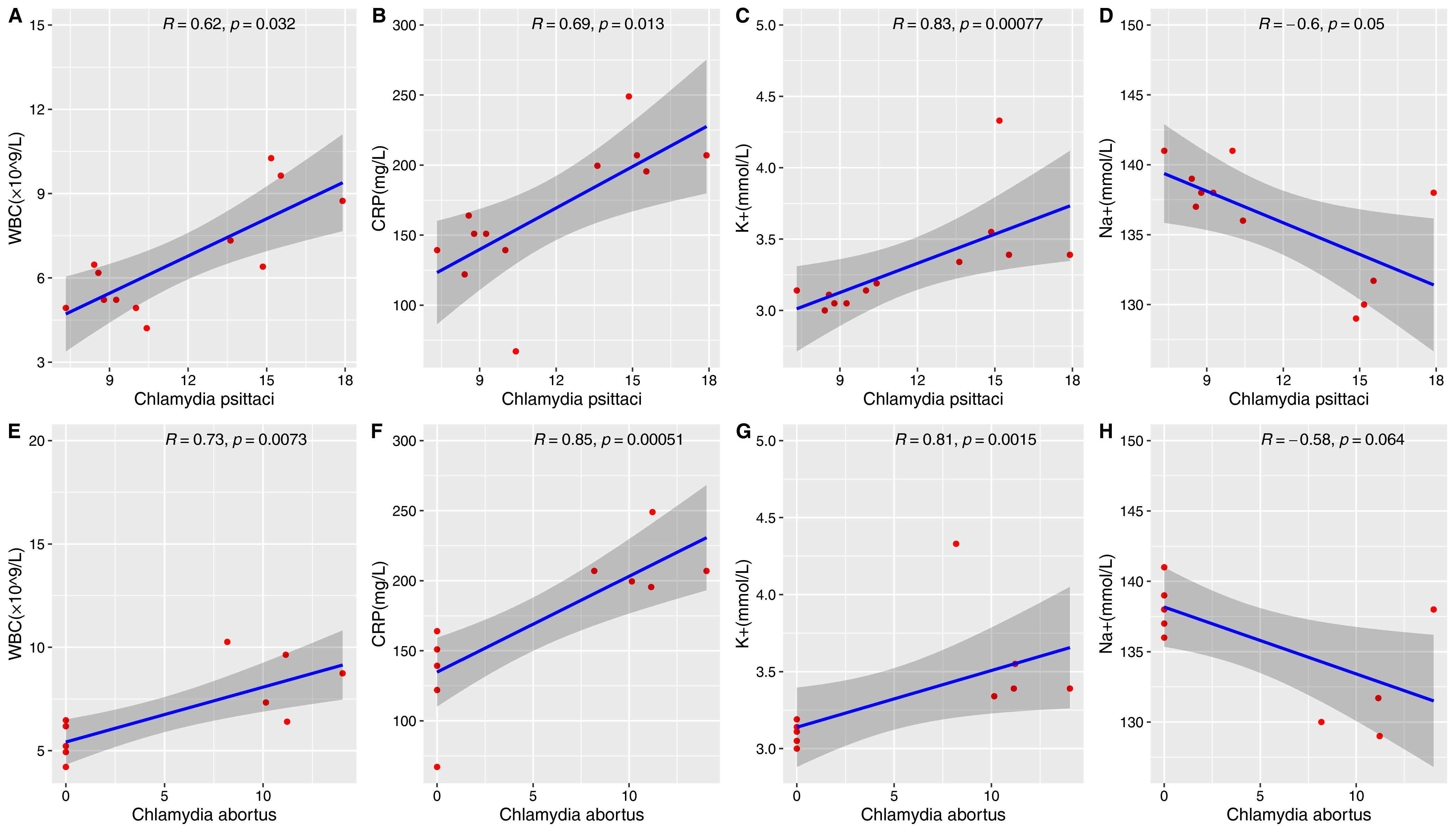

Supplement: Supplementary Figure 2 — Spearman’s test showed that Chlamydia psittaci was significantly positively correlated with WBC (A), CRP (B), K+ (C) and significantly negatively correlated with Na+ (D), and Chlamydia abortus was was significantly positively correlated with WBC (E), CRP (F), K+ (G) and negatively correlated with Na+ (H). [file Image_2.tif]

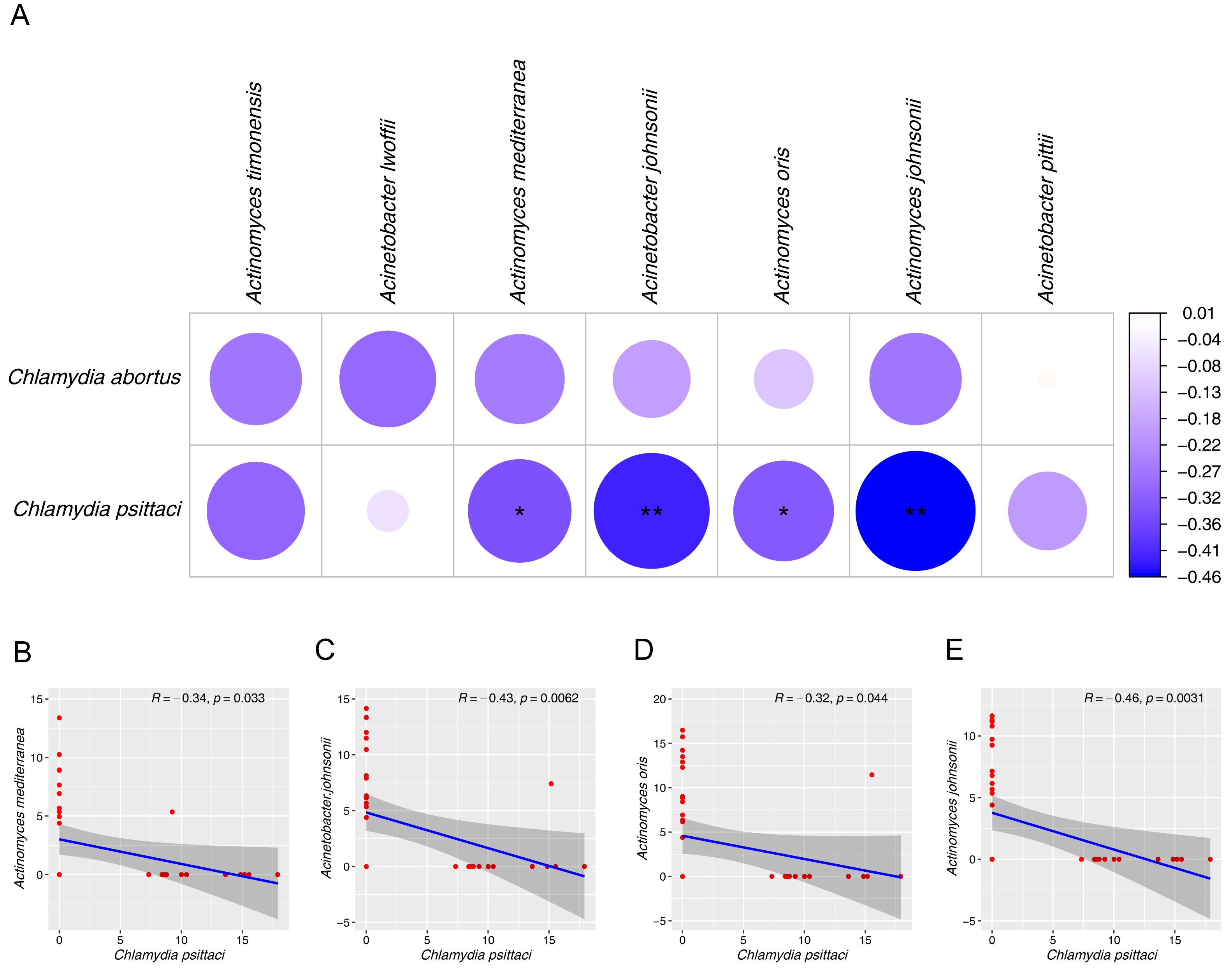

Supplement: Supplementary Figure 3 — Spearman correlation analysis between responsible pathogens with microbial species showed that Chlamydia psittaci was negatively correlated with micrbiobial species of Actinomyces mediterranea (A, B), Acinetobacter johnsonii (A, C), Actinomyces oris (A, D) and Actinomyces johnsonii (A, E). [file Image_3.tif]
